# Risk for renal injury from heat-related stress among outdoor workers and the imperative for climate-responsive health policies

**DOI:** 10.1016/j.joclim.2025.100600

**Published:** 2025-11-04

**Authors:** Leah Werner, Daniel Carrion, Nathalie Huguet

**Affiliations:** a Department of Family Medicine, Oregon Health & Science University, USA; b Environmental Health Sciences, Yale School of Public Health, USA

**Keywords:** Renal injury, Outdoor workers, Heat exposure, Climate change, Public health policy

## Abstract

Exposed to prolonged and extreme heat, outdoor workers face significant risks of acute and chronic kidney damage, including acute kidney injury and chronic interstitial nephritis in agricultural communities. Growing concerns over heat-related health issues in temperate, continental, and dry climates call for adaptive public health strategies that span diverse geographic areas. This communication reviews the policies in the United States addressing heat-related stress, identifies barriers to implementing effective health policies, and provides recommendations underscoring the need for comprehensive policy development to safeguard this high-risk population against climate-induced health threats.

## 1 Introduction

As climate change intensifies heat in temperate, continental, and dry zones, strengthening workplace protections, expanding preventive measures, and enhancing research rigor are critical. Proactive steps can protect vulnerable populations and build resilience for outdoor workers facing mounting heat threats. Heat stress is an “invisible but deadly” hazard, causing acute illnesses such as heat exhaustion, heatstroke, adverse cardiovascular events, acute kidney injury (AKI), and chronic kidney disease (CKD) from repeated dehydration and heat exposure [1,2]. While the exact burden in the United States (U.S.) is unknown, an estimated 15 % [3] of the 32 million outdoor workers [4]—approximately 4.8 million—experience heat-related illnesses annually. High-risk groups include agricultural laborers (such as farmers and migrant workers), roofers, construction crews, delivery drivers, highway maintenance and utility workers, and firefighters. Despite strong evidence, especially in agriculture and construction [1,2,5,6], the U.S. lacks comprehensive federal heat stress standards, relying on the Occupational Safety and Health Administration’s (OSHA) General Duty Clause, which results in inconsistent enforcement [7].

Federal efforts to address climate-related occupational health began under the Biden Administration, which elevated heat stress as a public health priority. However, these efforts were weakened by the current administration’s deregulation, reduced enforcement, and climate program rollbacks [8]. This erosion of federal capacity highlights the need for stronger state and local action. California, Washington, Oregon, and Colorado have adopted targeted standards, yet coverage nationwide remains uneven [[7], [8], [9], [10], [11]]. Barriers include limited policymaker awareness, inadequate enforcement funding, fragmented regulations, and industry resistance [8,[10], [11], [12], [13], [14]]. Addressing these requires enforceable climate-specific standards, research to identify vulnerable groups, and culturally appropriate worker education [14,15]. Collaborations among public health officials, labor, employers, and communities are essential to enforce protections and adapt strategies [9,15]. We assess U.S. heat safety policies, highlight implementation barriers, and propose solutions.

## 2 Existing U.S. heat safety policies

Federal and state policies that broadly address occupational heat stress are critical for preventing heat-related renal injury, including acute and chronic kidney diseases, among outdoor workers.

### 2.1 Federal policies

In the U.S., OSHA relies on the General Duty Clause, an insufficient tool for addressing emerging heat risks [16,17]. The clause obligates employers to provide workplaces free from hazards, including excessive heat, but the absence of a heat-specific standard has led to inconsistent enforcement [10,11]. To fill gaps, OSHA launched the National Emphasis Program (NEP), targeting high-risk workplaces; yet, these remain guidelines rather than binding rules [18]. The National Institute for Occupational Safety and Health (NIOSH) has also issued recommended standards, but like the NEP, they are advisory and lack legal force [19].

### 2.2 State policies

Several states have adopted heat protection standards to address federal gaps. California’s Heat Illness Prevention Standard requires training, water, shade, rest breaks, and acclimatization when temperatures exceed 80°F [20]. Washington and Oregon mandate similar outdoor protections, while Minnesota regulates indoor heat hazards [21]. Colorado recently enacted rules specifically for agricultural worker protection [22]. These initiatives reflect growing recognition of heat as an occupational hazard, but coverage remains uneven, with most states lacking specific standards.

## 3 Challenges to heat safety policies

U.S. climate health policies have shifted with each administration. Under Trump (2017–2021), worker protections suffered major setbacks amid a sweeping deregulatory agenda [22,23]. In 2017, OSHA halted rulemaking, freezing a proposed heat prevention standard mandating shade, hydration, and rest breaks [10,23,24]. Inspections for heat hazards declined even as heat-related injuries and deaths increased [24], and looming threats to OSHA’s capacity may further reduce its inspector workforce [24,25]. The administration also rescinded extended record-keeping and electronic injury reporting requirements, weakening hazard surveillance [24,26]. More broadly, federal agencies removed climate science references from public communications [23], including OSHA guidelines, reflecting an anti-science posture that reduced preparedness for climate-related occupational risks [10,13,24].

Public misinformation and politicized climate denial fueled skepticism, portraying even basic worker protections as government overreach and obstructing policy consensus [12]. Legal challenges during the Trump administration further constrained OSHA: a 2019 Review Commission ruling heightened the burden of proof for heat hazard violations, weakening the use of the General Duty Clause and discouraging enforcement [25]. Since January 2025, federal shifts have included the U.S. withdrawal from the Paris Agreement, rollbacks of domestic climate regulations, and termination of federal climate research funding. These actions reduce U.S. engagement in global initiatives and erode institutional capacity to address occupational heat risks, perpetuating preventable kidney injury as recurrent heat stress and dehydration accelerate renal decline.

## 4 Recommendations

With federal deregulation, state and local governments must adopt heat protections tailored to their Köppen–Geiger climate zones [26] and political contexts. The system classifies climates as tropical, arid, temperate, continental, or polar based on long-term patterns of temperature and precipitation. International practices demonstrate that such protections are feasible and effective. European nations mandate temperature thresholds for rest and hydration [8]; Middle Eastern countries enforce midday stoppages and temperature caps; and China requires regulated breaks with financial allowances [27]. The International Labour Organization calls for global standards, underscoring the U.S.’s delayed response [8]. These models reduce heat illness and offer pathways to mitigate acute and chronic kidney injury in U.S. workers.

The following recommendations outline an adaptive, decentralized approach to protecting workers from heat stress and related renal diseases:

1. Climate-Zone-Specific Adaptations: In arid and semi-arid regions (e.g., Southwest), states should adopt lower temperature thresholds for protection—hydration, shaded rest, and activity changes at cooler levels. Although evaporative cooling is efficient, it accelerates water loss, which can lead to dehydration [27,28]. Prolonged dry-heat exposure is linked to chronic kidney disease of nontraditional origin, underscoring dehydration as a renal hazard independent of hyperthermia [[29], [30], [31], [32], [33], [34], [35], [36]]. Acclimatization is necessary everywhere, but early action is crucial in arid zones where baseline heat approaches physiological limits. In humid regions (e.g., Southeast Asia), shaded rest, acclimatization, and frequent breaks are essential, as humidity impairs cooling and heightens renal risk [29]. In temperate zones with episodic heatwaves (e.g., Northeast, Pacific Northwest), states should adopt emergency plans including temporary work stoppages and renal monitoring [32,33].
2. Increased State and Local Regulatory Authority: States should amend preemption laws to allow local governments stronger heat protections. Florida’s House Bill 433 (July 2024) bars counties and cities from requiring employer protocols—such as water or rest breaks—beyond state or federal standards [37]. Texas’s 2023 “Death Star” law (House Bill 2127) likewise prohibits municipalities from enacting more protective workplace ordinances, including heat measures [38]. These statutes illustrate how preemption undermines tailored responses to renal health risks and highlight the urgency of preserving local flexibility. Enhanced authority would enable regionally specific policies responsive to climate and health conditions, improving effectiveness [11,24].
3. Robust Occupational Health Surveillance: State-level surveillance systems are needed to track occupational heat-related health outcomes. National systems, such as the Bureau of Labor Statistics’ injury survey and OSHA logs, rely on employer reports and rarely capture renal injuries. We recommend a proactive, integrated approach linking public health registries, hospital discharges, emergency department records, and workers’ compensation claims. Such integration would enable earlier detection of renal disease clusters, guide policy updates, support targeted interventions, and allow rapid responses to emerging threats [27,39].
4. Economic Feasibility and Political Viability: States and localities should frame protections around economic benefits, higher productivity, lower healthcare costs, and fewer workplace injuries. Practical heat standards can also reduce the long-term expenses of chronic kidney disease, including dialysis, transplants, and lost workforce participation. Highlighting these advantages can improve political acceptability and build broader stakeholder support. Proven policy frameworks should serve as models, with ongoing evaluation to ensure intended health and safety outcomes and allow adaptation as needed [10,24].
5. Model Policy Frameworks: Policymakers should draw from proven models like California’s standards mandating water, shade, and rest breaks [28]. States should also adopt international practices, including midday work stoppages in the Middle East and maximum working temperatures in the European Union, to protect workers across diverse climates [40,41]. These frameworks reduce dehydration and heat strain, key drivers of kidney injury in outdoor laborers.
6. Strengthened Labor Rights and Union Advocacy: States should strengthen unionization and collective bargaining rights, which improve compliance with safety standards, lower injury rates, and empower workers to secure protective measures from employers [42].
7. Comprehensive Worker and Employer Education: Mandated occupational health education should inform workers and employers about heat illness risks, preventive measures, and renal health impacts. Effective education can significantly reduce morbidity and mortality by enhancing early recognition and prompt response to heat-related illnesses [27].

These recommendations address the regulatory void left by federal deregulation with evidence-based, climate zone–specific strategies that empower local jurisdictions to protect worker health. Preventing heat-related renal injury requires a coordinated approach that unites science, policy, and community engagement. In the absence of strong federal action, states and municipalities can lead by adopting tailored protections, enhancing surveillance, and fostering cross-sector partnerships. International models demonstrate that rigorous heat standards are feasible and effective, and U.S. jurisdictions can adapt these lessons locally. Prioritizing viable, evidence-based interventions centered on vulnerable workers can reduce illness, preserve productivity, and build resilience.

## CRediT authorship contribution statement

**Leah Werner:** Writing – review & editing, Writing – original draft, Visualization, Validation, Supervision, Resources, Project administration, Methodology, Investigation, Formal analysis, Data curation, Conceptualization. **Daniel Carrion:** Writing – review & editing. **Nathalie Huguet:** Writing – review & editing.

## Declaration of competing interest

The authors declare that they have no known competing financial interests or personal relationships that could have appeared to influence the work reported in this paper.
